# Nucleotide excision repair pathway assessment in DNA exposed to low-intensity red and infrared lasers

**DOI:** 10.1590/1414-431X20154457

**Published:** 2015-07-10

**Authors:** A.S. Fonseca, V.M.A. Campos, L.A.G. Magalhães, F. Paoli

**Affiliations:** 1 Laboratório de Ciências Radiológicas, Departamento de Biofísica e Biometria, Instituto de Biologia Roberto Alcântara Gomes, Rio de Janeiro, RJ, Brasil; 2 Departamento de Ciências Fisiológicas, Instituto Biomédico, Universidade Federal do Estado do Rio de Janeiro, Rio de Janeiro, RJ, Brasil; 3 Centro de Ciências da Saúde, Centro Universitário Serra dos =rgãos, Teresópolis, RJ, Brasil; 4 Departamento de Morfologia, Instituto de Ciências Biológicas, Universidade Federal de Juiz de Fora, Juiz de Fora, MG, Brasil

**Keywords:** DNA, Enzyme, Escherichia coli, Filamentation, Laser

## Abstract

Low-intensity lasers are used for prevention and management of oral mucositis induced by anticancer therapy, but the effectiveness of treatment depends on the genetic characteristics of affected cells. This study evaluated the survival and induction of filamentation of *Escherichia coli* cells deficient in the nucleotide excision repair pathway, and the action of T_4_endonuclease V on plasmid DNA exposed to low-intensity red and near-infrared laser light. Cultures of wild-type (strain AB1157) *E. coli* and strain AB1886 (deficient in uvrA protein) were exposed to red (660 nm) and infrared (808 nm) lasers at various fluences, powers and emission modes to study bacterial survival and filamentation. Also, plasmid DNA was exposed to laser light to study DNA lesions produced *in vitro* by T_4_endonuclease V. Low-intensity lasers:*i*) had no effect on survival of wild-type *E. coli* but decreased the survival of uvrA protein-deficient cells,*ii*) induced bacterial filamentation, *iii*) did not alter the electrophoretic profile of plasmids in agarose gels, and*iv*) did not alter the electrophoretic profile of plasmids incubated with T_4_ endonuclease V. These results increase our understanding of the effects of laser light on cells with various genetic characteristics, such as xeroderma pigmentosum cells deficient in nucleotide excision pathway activity in patients with mucositis treated by low-intensity lasers.

## Introduction

Oral mucositis is a common inflammatory process caused by chemotherapy and radiotherapy against head and neck cancers (1). It can have severe effects on patient quality of life, including secondary infections, difficulty in swallowing and chewing, soreness, edema, erythema, ulcerations, bleeding, and pain during oncologic treatment (2). The strategies used to prevent and treat mucositis include basic oral care protocols, anti-inflammatory therapy, biologic response modifiers, cytoprotectants and cryotherapy (3). Use of low-intensity lasers to treat mucositis has gained in importance, and has been successful in prevention and management of oral mucositis induced by anticancer therapy (4).

Because of their biostimulatory effect, red and near-infrared lasers, which are nonthermal and nondestructive at low intensity, are widely used in a variety of health care settings for mucositis and other soft tissue repair. Biostimulation, an increase in cell metabolism following exposure to laser light, leads to alterations of biochemical reactions and changes in a number of cellular responses ([Bibr B05] [Bibr B06] 57). The responses to low-intensity laser irradiation are related to alterations of the regenerative potential of tissues, neovascularization, and formation of scar tissue (8,9). Cytochrome C in mammalian cells and cytochrome BD in bacterial cells are considered to be chromophores involved in the absorption of red and near-infrared laser light (10,11). A primary photosignal stimulated by absorption of laser energy is subsequently transduced to an amplified signal in the cells (10). Highly reactive chemical compounds are generated in these transduction-signal pathways, and they have potential photobiological side effects (5). In fact, it has been suggested that laser radiation induces free radical production (5,12,13), which could produce an imbalance between oxidant and antioxidant concentration. An excess of free radicals, by reacting with biomolecules, could cause modifications of cellular function, leading to undesired effects. The nature of laser-induced side effects is still in dispute, although there are data about DNA damage after laser exposure in eukaryotic ([Bibr B13] [Bibr B14] 1315) and prokaryotic cells (16,17). Moreover, previous studies have demonstrated that red and near-infrared lasers decrease survival of *E. coli* cells deficient in base excision repair, a pathway involved in repair of oxidative DNA lesions, and induce the filamentation phenotype more frequently than in wild type *E. coli*cells (18-20). While the controversies about laser-induced DNA lesions by free radicals have not been resolved, it is interesting to evaluate whether other DNA repair pathways are involved in cellular responses to low-intensity lasers.

Nucleotide excision repair is an important DNA repair pathway involving excision of pyrimidine dimers and other bulky lesions induced in DNA by ultraviolet radiation. In *E. coli*, this pathway comprises three proteins (uvrA, uvrB, and uvrC) that have recognition, cleavage, and endonuclease functions. Compared with "excision-repair proficient" wild-type cells, *E. coli*cells deficient in one or more of these genes are more sensitive to ultraviolet radiation, and were used in early studies to simulate xeroderma pigmentosum cell responses to solar ultraviolet radiation (21). Expression of the *uvr* gene comprises part of a response set dominated by the SOS function in bacterial cells exposed to hazardous physical and chemical agents (22). In addition to *uvr*gene expression, filamentation, anomalous bacterial growth with cell elongation in the absence of septa formation, occurs in response to ultraviolet radiation (23). This morphological abnormality has been used to evaluate effects of environmental agents on the integrity of DNA in bacterial cells (24).

With the increasing use of low-intensity lasers for treating various diseases, possible adverse effects on DNA must be considered. This is particularly important in the case of laser-based treatment of oral cavity disease, where the occurrence of radiation-induced mucositis, a potential side effect, has not been clearly documented or evaluated. This study evaluated survival and filamentation induction in *E. coli* cells deficient in the nucleotide excision repair pathway, and the action of T4 endonuclease V on plasmid DNA exposed to low-intensity red and near-infrared laser light. Various laser fluences, powers, and frequencies were selected from those described in the laser device manual.

## Material and Methods

### Low-intensity red and near-infrared lasers and chemical reagents

A therapeutic low-intensity red (InGaAIP) and near-infrared (GaAlAs) laser (100 mW; Photon Lase III), with emissions of 660 and 808 nm, was purchased from D.M.C Equipamentos Ltda. (Brazil). Ethidium bromide, xylene cyanol, bromophenol blue, glycerol, agarose, tris(hydroxymethyl)aminomethane (tris), and ethylenediamine tetraacetic acid (EDTA) were from Merck (USA). Sodium chloride (NaCl), acetic acid, and sodium hydroxide (NaOH) were from Vetec (Brazil). DNA repair enzyme from *E. coli* (T_4_ endonuclease V) was from New England Biolabs (USA).

### Survival of *E. coli* cells

Cultures of *E. coli* AB1157 (wild-type) and AB1886 (uvrA deficient) in the exponential growth phase (10^8^ cells/mL, 2-3 h, 37°C) were grown from stocks in the stationary growth phase. The cells were collected by centrifugation (700 *g*, 15 min), washed twice in saline (0.9% NaCl), and resuspended in saline. Aliquots (20 µL, n=5, for each fluence, power and emission mode) of bacterial suspensions (10^8^cells/mL) were exposed, at room temperature and under white light (fluorescent lamps), to low-intensity red and near-infrared laser light (0.00785 cm^2^ beam diameter) at various fluences (25, 50, and 100 J/cm^2^, corresponding to 0.7, 1.5, and 2.8 J, respectively), and powers (30, 50, and 100 mW) in continuous wave and pulsed emission modes (10, 30, and 100 pulses/s). The laser source was positioned very close to the surface of the bacterial suspension, which was covered by the beam. The tissue exposure time was automatically adjusted by the laser device as a function of power and fluence (e.g., 7, 14, and 28 s for 25, 50, and 100 J/cm^2^, respectively). Bacterial suspensions not exposed to lasers were used as controls. Bacterial suspensions were diluted in saline, plated onto*Petri* dishes containing solidified rich medium (Luria-Bertani, 1.5% agar). Colonies that formed following overnight incubation at 37°C were counted, and the survival fractions were calculated (25). Experiments were performed in triplicate, and results are reported as the mean of three independent assays.

### *In vitro* DNA repair enzyme activity on plasmid DNA

T_4_ endonuclease V from *E. coli* was used in an*in vitro* DNA repair assay to evaluate the effect of low-intensity red and infrared laser light. Aliquots of plasmid DNA were exposed, at room temperature and in the light, to low-intensity red and infrared lasers as described in the bacterial survival assay. Immediately afterward, plasmids (approximately 200 ng) were mixed with enzyme buffer (2 units) and incubated (37°C, 30 min). Each sample was then mixed with loading buffer (0.25% xylene cyanol, 0.25% bromophenol blue, and 25% glycerol in water) and applied onto a horizontal 0.8% agarose gel electrophoresis chamber in tris-acetate-EDTA buffer (40 mM tris, 20 mM acetic acid, 1 mM EDTA, pH 8.0, 7 V/cm). Gels were stained with ethidium bromide (0.5 µg/mL), and the plasmid forms were viewed under fluorescence using an ultraviolet transillumination system. The assay was repeated at least three times. Digital images of the gels and plasmid forms were analyzed semiquantitatively using the ImageJ for Windows software (http://rsb.info.nih.gov/ij/index.html).

#### Bacterial filamentation assay and morphological measurements

Exponential growth phase cultures of *E. coli* strains AB1157 and AB1886 (10^8^ cells/mL) were grown and exposed to low-intensity red and infrared laser light as described in the bacterial survival assay. Bacterial suspensions not exposed to laser light were used as controls. Immediately after laser exposure, aliquots (10 µL) were spread onto microscopic slides and Gram-stained (26). Bacterial cells were visualized with a light microscope (Carl Zeiss, Germany) equipped with an A-plan 40× objective, 0.90 condenser and 100 W halogen lamp. The images were captured with an AxioCam HRc Sony 12M color microscopy camera (Carl Zeiss), using the Zeiss Axiovision software (http://www.zeiss.com/microscopy/en_us/products/microscope-software/axiovision-for-biology.html). The images were analyzed using the Image-Pro Plus 6.0 software for Windows XP (MediaCybernetics, USA) to determine the percentage of bacterial filamentation. A bacterial filament was considered to have an area 2.5 times larger than the mean bacterial cell area. Experiments were carried out in duplicate, and the results are reported as the means of three independent assays.

#### Statistical analysis

Data are reported as means±SD of the bacterial survival fractions, plasmid forms, and the percentages, area, and perimeter of bacterial filaments. One-way analysis of variance (ANOVA) was performed to determine the significance of differences in the reported results, followed by the Bonferroni post-test, with P<0.05 as the significance level. The InStat Graphpad software (GraphPad InStat for Windows XP, USA) was used to perform the statistical analysis.

### Results

#### Survival of *E. coli* cultures exposed to red and infrared lasers

Table 1 shows the survival fractions of *E. coli* AB1157 and AB1886 cultures exposed to low-intensity red and infrared lasers at various fluences. No significant effect (P&0.05) on survival was observed following irradiation of*E. coli* AB1157 cultures with red or infrared lasers. However, survival fractions of AB1886 cultures exposed to the red laser at the highest fluence (100 J/cm^2^) and to the infrared laser at all fluences used were significantly decreased (P<0.05).

**Table:**
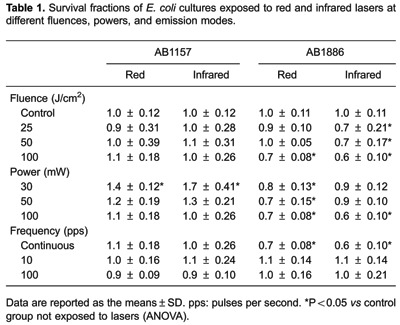


To ascertain whether the laser beam power had an effect on laser-induced biological effects, survival was determined in *E. coli*AB1157 and AB1886 cultures exposed to red and infrared lasers at increasing powers, with the highest fluence being 100 J/cm^2^(Table 1). The survival fraction was significantly greater in *E. coli* AB1157 cultures exposed to both red and infrared lasers at the lowest power (30 mW) than it was in cultures exposed at the highest power (100 mW, P<0.05). On the other hand, the survival fraction in *E. coli* AB1886 cultures was significantly decreased only in those exposed to the infrared laser at 100 mW (P<0.05).

The emission mode (continuous wave and pulsed laser light) was also evaluated in this study. *E. coli* cultures were exposed to red and infrared lasers in the continuous wave and pulsed emission (at 10 and 100 pps) modes at the highest fluence (100 J/cm^2^) and power (100 mW), and the survival fractions were evaluated (Table 1). Survival of *E. coli* AB1157 was not dependent on the laser emission mode; no significant (P&0.05) differences in survival fraction were observed. However, survival of exposed*E. coli* AB1886 cultures was dependent on the emission mode. The continuous wave mode caused a significant (P<0.05) decrease of the survival fraction, but the pulsed mode did not cause significant (P&0.05) alterations of bacterial survival in response to either red or infrared laser emissions.

#### DNA repair enzyme activity on plasmid DNA exposed to low-intensity red and infrared lasers

**Figure 1:**
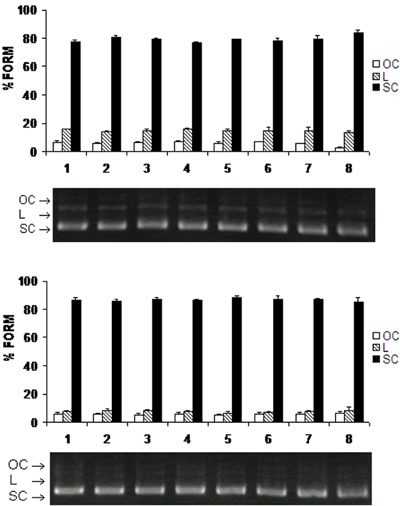
Percentages of bacterial plasmid forms and photographs of agarose gels after electrophoresis of plasmid pUC19 exposed to red lasers (*top*) and to infrared lasers (*bottom*), at 100 mW in continuous wave emission mode, and incubated with T4 endonuclease V.*Top*:*lane 1*, pUC19;*lane 2*, pUC19+T4 endonuclease V; *lane 3*, pUC19+red laser 25 J/cm^2^; *lane 4*, pUC19+red laser 25 J/cm^2^+T4 endonuclease V;*lane 5*, pUC19+red laser 50 J/cm^2^;*lane 6*, pUC19+red laser 50 J/cm^2^+T4 endonuclease V; *lane 7*, pUC19+red laser 100 J/cm^2^; *lane 8*, pUC19+red laser 100 J/cm^2^+T4 endonuclease V.*Bottom*:*lane 1*, pUC19;*lane 2*, pUC19+T4 endonuclease V;*lane 3*, pUC19+infrared laser 25 J/cm^2^; *lane 4*, pUC19+infrared laser 25 J/cm^2^+T4 endonuclease V; *lane 5*, pUC19+infrared laser 50 J/cm^2^;*lane 6*, pUC19+infrared laser 50 J/cm^2^+T4 endonuclease V;*lane 7*, pUC19+infrared laser 100 J/cm^2^;*lane 8*, pUC19+infrared laser 100 J/cm^2^+T4 endonuclease V. Data are reported as the means±SD for n=3 independent experiments. OC: open circle, L: linear, SC: supercoiled.

Figure 1 shows photographs of agarose gels and graphs of the percentages of bacterial plasmid forms quantified by electrophoresis after exposure to low-intensity red (Figure 1 top) and infrared (Figure 1 bottom) lasers at various fluences and incubated with T_4_ endonuclease V. Data in this figure indicate that exposure to lasers did not significantly (P&0.05) induce quantitative or qualitative alterations in the electrophoretic profile of plasmid DNA as was determined in preliminary experiments (data not shown). The electrophoretic profiles of plasmids exposed to red or infrared lasers and incubated with T_4_ endonuclease V were not significantly (P&0.05) different from profiles of plasmids not incubated with T_4_ endonuclease V.

**Figure 2:**
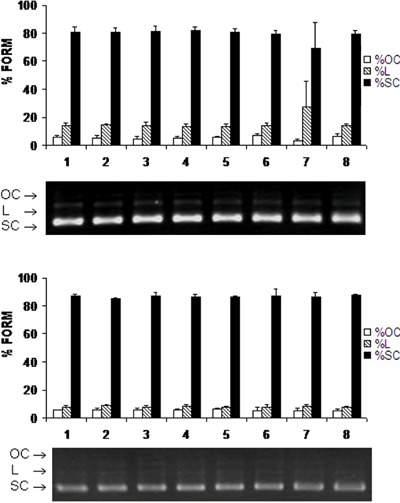
Percentages of bacterial plasmid forms and photographs of agarose gels after electrophoresis of plasmid pUC19 exposed to red lasers (*top*) and to infrared lasers (*bottom*) at different powers, 100 J/cm^2^ in continuous wave emission mode, and incubated with T4 endonuclease V. *Top*: *lane 1*, pUC19; *lane 2*, pUC19+T4 endonuclease V; *lane 3*, pUC19+red laser 30 mW;*lane 4*, pUC19+red laser 30 mW+T4 endonuclease V; *lane 5*, pUC19+red laser 50 mW;*lane 6*, pUC19+red laser 50 mW+T4 endonuclease V; *lane 7*, pUC19+red laser 100 mW;*lane 8*, pUC19+red laser 100 mW+T4 endonuclease V. *Bottom*: *lane 1*, pUC19;*lane 2*, pUC19+T4 endonuclease V;*lane 3*, pUC19+infrared laser 30 mW;*lane 4*, pUC19+infrared laser 30 mW+T4 endonuclease V; *lane 5*, pUC19+infrared laser 50 mW; *lane 6*, pUC19+infrared laser 50 mW+T4 endonuclease V; *lane 7*, pUC19+infrared laser 100 mW; *lane 8*, pUC19+infrared laser 100 mW+T4 endonuclease V. Data are reported as the means±SD for n=3 independent experiments. OC: open circle, L: linear, SC: supercoiled.

To determine whether the power of the laser beam influenced the DNA repair enzyme activity, plasmids were exposed to lasers at 100 J/cm^2^ in continuous wave emission mode at various powers. No significant (P&0.05) quantitative or qualitative alterations in the electrophoretic profiles of plasmids were observed after exposure to either red or infrared lasers. Similar results were obtained when plasmids were exposed to lasers and incubated with T_4_ endonuclease V (Figure 2).

**Figure 3:**
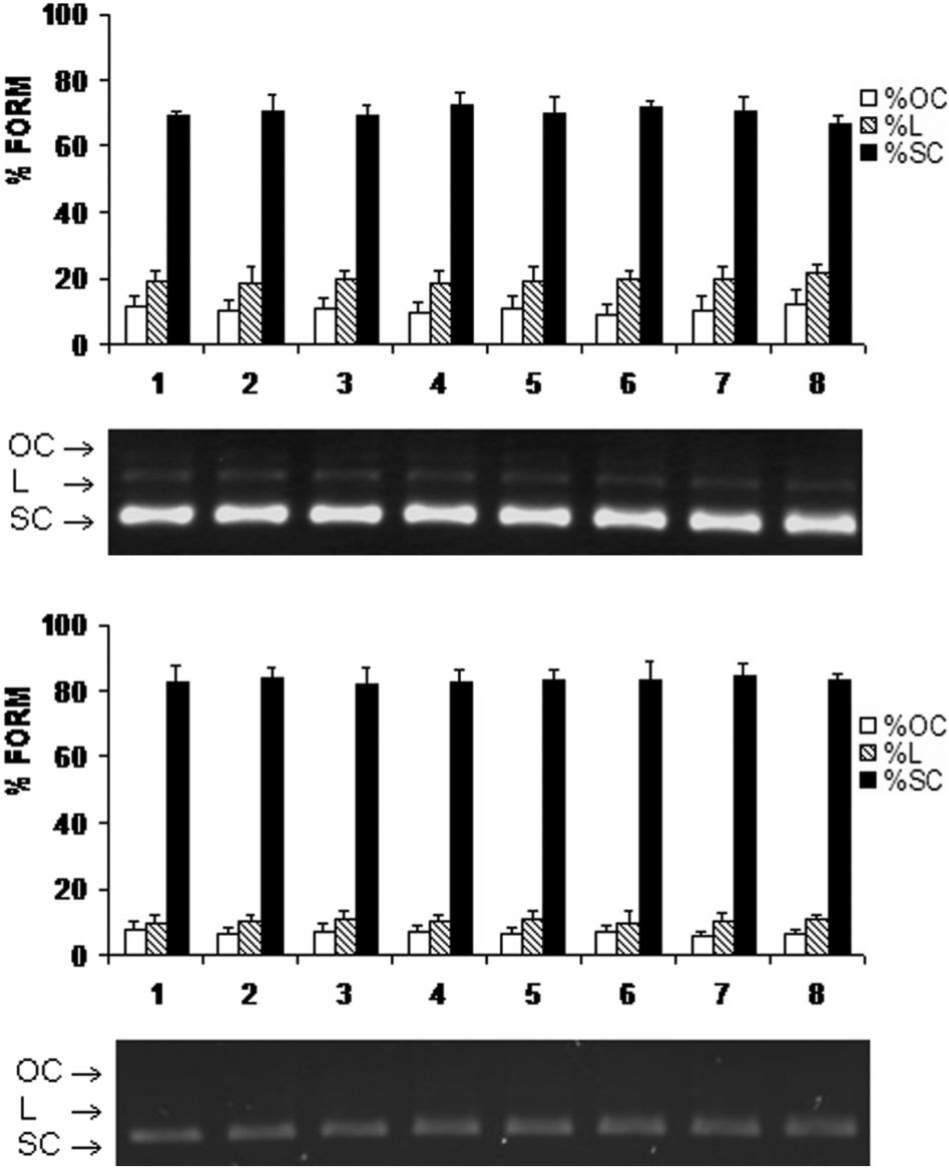
Percentages of bacterial plasmid forms and photographs of agarose gels after electrophoresis of plasmid pUC19 exposed to red lasers (*top*) and to infrared lasers (*bottom*), at different frequencies, 100 mW and 100 J/cm^2^, and incubated with T4 endonuclease V.*Top*: *lane 1*, pUC19;*lane 2*, pUC19+T4 endonuclease V;*lane 3*, pUC19+red laser 10 pps;*lane 4*, pUC19+red laser 10 pps+T4 endonuclease V;*lane 5*, pUC19+red laser 30 pps; *lane 6*, pUC19+red laser 30 pps+T4 endonuclease V;*lane 7*, pUC19+red laser 100 pps; *lane 8*, pUC19+red laser 100 pps+T4 endonuclease V.*Bottom*: *lane 1*, pUC19;*lane 2*, pUC19+T4 endonuclease V;*lane 3*, pUC19+infrared laser 10 pps;*lane 4*, pUC19+infrared laser 10 pps+T4 endonuclease V; *lane 5*, pUC19+infrared laser 30 pps; *lane 6*, pUC19+infrared laser 30 pps+T4 endonuclease V;*lane 7*, pUC19+infrared laser 100 pps;*lane 8*, pUC19+infrared laser 100 pps+T4 endonuclease V. Data are reported as the means±SD for n=3 independent experiments. OC: open circle, L: linear, SC: supercoiled.

Significant alterations were not seen in the electrophoretic profiles of plasmids exposed to red or infrared lasers, or in plasmids exposed to lasers and incubated with the enzyme in different emission modes (continuous wave or pulsed) with a fluence of 100 J/cm^2^ and power of 100 mW (Figure 3).

#### Bacterial filamentation in *E. coli* cultures exposed to red and infrared lasers

**Figure 4:**
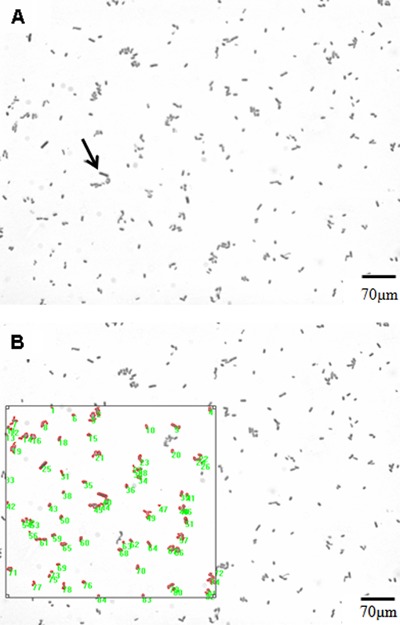
Representative bacterial filamentation images from AB1157 cultures in exponential growth phase. *A*, The arrow indicates a bacterial filament; *B*, Same image demonstrating how image analysis was performed. A bacterial filament was considered to have an area 2.5 times larger than the mean bacterial cell.

Figure 4 shows representative images of cells from *E. coli* AB1157 cultures in exponential growth phase (Figure 4A) and analysis of induction of bacterial filaments (Figure 4B). Table 2 shows the percentages of bacterial filaments in cultures of *E. coli*AB1157 and AB1886 exposed to low-intensity red and infrared lasers at different fluences, powers, and emission modes. Laser exposure significantly (P<0.05) increased the percentage of bacterial filaments in both*E. coli* AB1157 and AB1886 cultures. Filamentation induction was fluence-dependent in *E. coli* AB1157 for the infrared laser and inversely fluence-dependent in *E. coli*AB1886 for the red laser. Also, at the lowest powers, a significant (P<0.05) increase in the percentage of bacterial filaments occurred in*E. coli* AB1157 cultures exposed to lasers at 100 J/cm^2^ in the continuous wave emission mode. However, at low power (30 mW), neither the red nor the infrared laser significantly increased the percentage of bacterial filaments in *E. coli*AB1886 cultures (P&0.05). Lasers significantly (P<0.05) increased the percentage of bacterial filaments in *E. coli*AB1157 and AB1886 cultures in both continuous wave and pulsed emission modes. However, this effect was not dependent on pulse frequency, except for the red laser in *E. coli* AB1886.

**Table:**
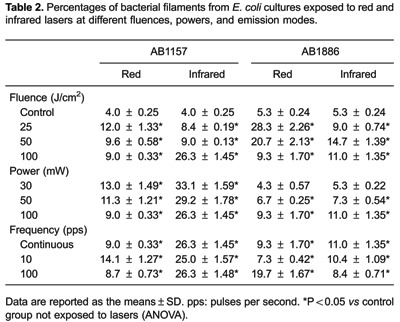


#### Evaluation of morphology of *E. coli* cells exposed to red and infrared lasers

Area and perimeter of *E. coli* AB1157 and AB1886 cells were evaluated following exposure to red and infrared lasers at different fluences, powers, and emission modes (Tables 3 and 4). At 100 mW in the continuous wave emission mode, red laser exposure significantly (P<0.05) reduced *E. coli* AB1157 cell area at the highest fluence evaluated (100 J/cm^2^). On the other hand, the infrared laser significantly (P<0.05) increased both area and perimeter of those bacterial cells. When the cells were exposed to the red laser at lower powers (30 and 50 mW) at 100 J/cm^2^ in continuous wave emission, no significant (P&0.05) alteration in area and perimeter occurred. However, under the same conditions, the infrared laser induced increases in both area and perimeter of *E. coli* AB1157 cells. Similar to the results obtained at lower powers, the red laser in pulsed emission mode, 100 mW, and 100 J/cm^2^ did not induce area and perimeter changes of *E. coli* AB1157 cells, but the infrared laser induced significant (P<0.05) increases of both morphological parameters. Area and perimeter were also evaluated in *E. coli* AB1886 following exposure to both red and infrared lasers (Table 4) under the same conditions evaluated for *E. coli* AB1157 cells. No significant changes (P&0.05) in either morphological parameter were observed in *E. coli*AB1886 cells following exposure to either red or infrared lasers at 100 mW in continuous wave mode emission or at any of the tested fluences. However, laser exposure at lower powers (30 and 50 mW), 100 J/cm^2^, and continuous wave emission, significantly (P<0.05) decreased area and perimeter of these cells. In pulsed emission mode, both lasers induced a decrease of morphological parameters, except for the red laser at 100 pps.

**Table:**
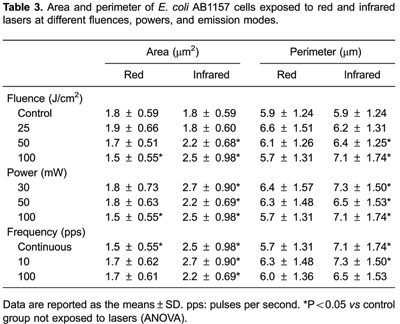


**Table:**
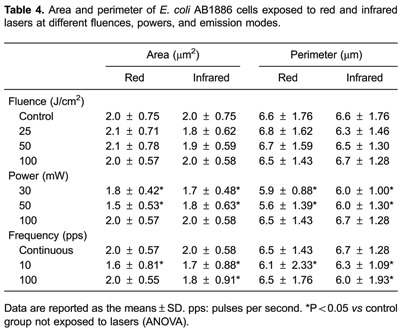


### Discussion

Some studies have suggested that exposure to low-intensity laser light induces cellular alterations causing increase in resistance to nonionizing (27), ionizing (28), and oxidative chemical compounds (25). Those studies prompted evaluation of the effects of lasers on DNA molecules (1820,). Our study results show that low-intensity red lasers at fluences, powers, and emission modes used in clinical protocols were not lethal to *E. coli* cells proficient in DNA repair (strain AB1157, Table 1). Instead, survival fractions were increased at the lowest power (30 mW), suggesting a power-dependent biological effect (biostimulation). Previous studies demonstrated a biostimulatory effect in prokaryote (10) and eukaryote (30) cells. Recently, we observed this effect in cell cultures proficient in DNA repair mechanisms, but not in an*E. coli* strain deficient in repair of oxidative DNA lesions (20). No studies have demonstrated biostimulation that was dependent on laser beam power. Here, survival of uvrA-deficient *E. coli* cells was decreased by red lasers at all evaluated powers, but only at the highest fluence (100 J/cm^2^) and in the continuous wave emission mode. These results agree with previously reported data, suggesting that the biological effects of low-intensity lasers are frequency-dependent (25). Moreover, these cells were sensitive to infrared lasers at lower fluences, but only at the highest power (100 mW) and in the continuous wave emission mode. Despite the importance of this biological aspect, our study describes, for the first time, how cells deficient in the nucleotide excision repair of DNA damage are sensitive to low-intensity laser radiation. These results support the hypothesis that laser-induced biological effects are dependent on DNA repair mechanisms (17).

T_4_ endonuclease V is a highly specific glycosylase that cleaves the glycosydic bond of cyclobutane pyrimidine dimers induced by ultraviolet radiation and possesses a concomitant DNA apurinic or apyrimidinic (AP) lyase activity at basic sites generated by glycosylase action (31). Due to these properties, T_4_ endonuclease V protects patients with xeroderma pigmentosum against skin cancer (32). Plasmid DNA exposed to low-intensity red and infrared lasers did not show any alterations in electrophoretic profile in agarose gels, suggesting absence of detectable single- or double-strand DNA breaks, as previous studies have demonstrated (19,20). In addition, the plasmids did not show any alteration in their electrophoretic profile after incubation with T_4_ endonuclease V, suggesting that red and infrared laser lights did not induce DNA lesions targeted by this enzyme, at least under the laser irradiation conditions used in this study.

Whether significant laser light energy is directly or indirectly absorbed by DNA molecules remains controversial. For ultraviolet A radiation (320-400 nm), it has been suggested that DNA lesions are induced by direct (33) or indirect mechanisms (34). An unidentified chromophore might be involved in the indirect mechanism for absorbing the ultraviolet radiation energy and subsequently transferring it to DNA molecules (34). A similar indirect mechanism might be related to absorption of laser light energy by cells and its effects on the DNA molecule. In fact, cytochrome C in eukaryotic and cytochrome BD in prokaryotic cells are chromophores for red and infrared laser light (11). This could explain the lack of change in the electrophoretic profile of plasmids incubated with T_4_ endonuclease V, even though *E. coli* AB1886 cultures were sensitive to red and infrared lasers.

Previous studies have demonstrated induction of filamentation in *E. coli* cells exposed to low-intensity lasers ([Bibr B18] [Bibr B19] 1820). Wild-type *E. coli*cells (strain AB1157) exhibited a filamentous phenotype after exposure to both red and infrared lasers, and primarily with infrared lasers at the highest fluence (100 J/cm^2^). Interestingly, laser-induced filamentation was slightly greater at lower powers, but was similar for the continuous wave and pulsed emission modes, except at 10 pps for the red laser. In this study, the filamentous phenotype was described for the first time in a strain of *E. coli* deficient in nucleotide excision repair (strain AB1886, Table 2). Except for red laser exposures at low fluences (25 and 50 J/cm^2^), *E. coli* AB1886 cells exhibited the lowest percentages of bacterial filaments. This can be attributed to increased cellular inactivation at those fluences (Table 1), preventing more cells from presenting a filamentous phenotype. In fact, cellular filamentation is a strategy to restore normal internal conditions, mainly DNA lesions, in order to survive injury (24). The appearance of red and infrared laser-induced filamentous phenotypes was power-, but not frequency-dependent, occurring after exposure to lasers in the continuous wave and pulsed emission modes.

The filamentation assay performed in this study showed that low-intensity red and infrared lasers induced SOS responses in bacterial cells. However, the assay permitted evaluation of the percentage of bacterial filaments induced by laser exposure but not the morphology of cells that did not present that phenotype. Computer software has been used to perform measurements of morphological parameters (35). In fact, data in Table 2 show that bacterial filaments occurred in fewer than 30% of both *E. coli* AB1157 and AB1886 cultures exposed to lasers.

Cell area and perimeter were measured to evaluate the morphology of bacterial cells that did not exhibit a filamentous phenotype. Red and infrared laser light induced different alterations in *E. coli* AB1157 cell area and perimeter (Table 3). Red laser exposure decreased cell area at the highest fluence (100 J/cm^2^) and power (100 mW) in the continuous wave emission mode. Infrared laser exposure increased both morphological parameters at 50 J/cm^2^, at the lower powers, and in both the continuous wave and pulsed emission modes. These results demonstrate that the wavelength of laser light acts to cause changes in cell morphology. In*E. coli* AB1886 cells, red and infrared lasers at the highest power in the continuous wave mode did not alter cell morphology (Table 4). However, at lower powers and in the pulsed emission mode, both red and infrared lasers altered the area and perimeter of cells, but both lasers decreased those parameters. These results reinforce the suggestion that biological effects induced by low-intensity lasers depend on physical parameters (power and emission mode) as well as the presence of DNA repair mechanisms (17). On the other hand, data from morphological analysis indicate that low-level laser light can alter the function of membrane ion channels. Changes in morphological parameters such as area and perimeter are associated with changes in the function of those membrane proteins (36). In fact, He-Ne lasers operating at 632.8 nm increase or decrease the amplitude of membrane potentials dependent on slow potassium currents in a fluence-dependent way (37). Low pulse energy neodymium:yttrium-aluminum-garnet (Nd:YAG) laser light increases intracellular Ca^2+^ concentration in osteoblasts through the activation of TRPC1 ion channels (38). Membrane conductance through voltage-gated K^+^, BK, and Kir channels and T- and L-type Ca^2+^ channels is increased following red laser exposure at low fluence and power (39). Also, infrared lasers at 810 nm raise mitochondrial membrane potential and reduce intracellular Ca^2+^ concentration (40).

Although our research was performed with bacterial cells, low-intensity lasers at fluences used in clinical protocols could induce biological effects depending on cellular genetic characteristics, such as functional DNA repair by the nucleotide excision pathway.
